# Bilirubin adsorption versus plasma exchange for hyperbilirubinemia in patients after cardiac surgery: a retrospective study

**DOI:** 10.1186/s13019-021-01622-8

**Published:** 2021-08-23

**Authors:** Ke Pan, He Zhang, Kai Zhong, Hai-tao Zhang, Ze-shi Li, Zhong Chen, Su-ping Gu, Man Xie, Tuo Pan, Hai-long Cao, Dong-jin Wang

**Affiliations:** 1 Department of Cardio-Thoracic Surgery, Nanjing Drum Tower Clinical College of Xuzhou Medical University, Xuzhou, China; 2 Department of Cardio Thoracic Surgery, Nanjing Drum Tower Hospital, Chinese Academy of Medical Science and Peking Union Medical College, Graduate School of Peking Union Medical College, Beijing, China; 3 Department of Cardio-Thoracic Surgery, Nanjing Drum Tower Hospital Clinical College of Nanjing Medical University, Number 321 Zhongshan Road, Jiangsu, 210008 China; 4 Department of Cardio-Thoracic Surgery, Nanjing Drum Tower Hospital, The Affiliated Hospital of Nanjing University Medical School, Number 321 Zhongshan Road, Jiangsu, 210008 China

**Keywords:** Hyperbilirubinemia, Bilirubin adsorption, Plasma exchange, Cardiac surgery

## Abstract

**Objective:**

Hyperbilirubinemia after cardiac surgery increases in-hospital mortality and is associated with poor prognosis. Our present study aimed to compare the efficacy of bilirubin adsorption (BA) and plasma exchange (PEX) in patients with hyperbilirubinemia after cardiac surgery.

**Methods:**

We retrospectively included patients who underwent BA treatment or PEX treatment due to severe hyperbilirubinemia after cardiac surgery at our center from 2015 to 2020. We collected results from urine and liver function tests before and after treatment and compared the in-hospital mortality and morbidity between the two treatment groups.

**Results:**

A total of 56 patients were enrolled in this study: 14 patients received BA treatment, and 42 patients received PEX treatment. Compared to the PEX group, the BA group exhibited a statistically significant reduction in total bilirubin (p = 0.016) and direct bilirubin (p = 0.036) levels. The in-hospital mortality was 85.7% (48/56) in the whole group, and the BA group had a lower mortality than the PEX group (71.4% vs. 90.5%, p = 0.078). The BA group showed better circulatory support, including lower risks of IABP (21.4% vs. 52.4%, p = 0.044), ECMO (21.4% vs. 50.0%, p = 0.061), reintubation (64.3% vs. 40.5%, p = 0.122) and ventricular arrhythmias (64.3% vs. 45.2%, p = 0.217). The in-hospital mortality was still lower in the BA treatment group than in the PEX treatment group (71.4% vs. 100%, p = 0.049) in the matched cohort.

**Conclusions:**

Compared to PEX treatment, BA treatment had a higher bilirubin removal ability in patients with hyperbilirubinemia and could reduce the mortality and risks of poor clinical outcomes. BA treatment should be considered an effective treatment method for patients with higher total bilirubin or direct bilirubin levels.

## Introduction

Cardiac surgery with cardiopulmonary bypass (CPB) can lead to different degrees of liver damage. In a previous series of reports, the rate of hyperbilirubinemia was reported to be approximately 8.6% to 40% [1–3]. Hyperbilirubinemia significantly increases the risk of mortality and morbidity [4]. The most serious level of liver damage is acute liver failure (ALF). ALF usually develops into multiple organ dysfunction syndrome (MODS) after cardiac surgery. The rate of MODS combined with ALF is relatively low (4.7%), while it could increase the mortality rate up to 90% [5, 6].

Current treatments for ALF or hyperbilirubinemia include plasma exchange (PEX), molecular adsorbent recirculating system (MARS), extracorporeal blood purification and bilirubin adsorption (BA). All of these treatment methods are based on their ability to remove endotoxin, cytokines and bilirubinemia from blood, thereby functioning in detoxification and creating conditions for liver cell regeneration.

PEX is a nonbiological artificial liver support system and has been an effective method for treating ALF. PEX treatment is recommended for the early stage of viral hepatitis and liver failure. BA is an important part of the artificial liver support system to overcome adverse reactions such as plasma dosage restrictions, plasma allergies and blood transfusion infection. It is another option for the treatment of ALF and hyperbilirubinemia.

Multiple processes are associated with ALF in patients after cardiac surgery, including CPB, low cardiac output syndrome and elevation of venous pressure. Therefore, it remains controversial which treatment for ALF after cardiac surgery has better clinical outcomes. Furthermore, only a few clinical studies with limited sample sizes have been performed to address this question. PEX and BA have been used at our center for the treatment of ALF after cardiac surgery since 2015. We designed this retrospective study to compare the effectiveness of BA and PEX treatment in patients with ALF after cardiac surgery.

## Methods

### Study population

From 2015 to 2020, 11,483 adult patients underwent cardiac surgery at our center, 56 of whom were diagnosed with AFL or hyperbilirubinemia after cardiac surgery. Fourteen patients who received BA treatment were chosen from 2019 to 2020, and forty-two  patients who received PEX treatment were chosen from 2015 to 2020. This study was approved by the Medical Ethics Committee of Affiliated Nanjing Drum Tower Hospital, Nanjing University Medical College (2020–249-01). The requirement for informed consent from the patients was waived, and all the authors had full control of the data and information regarding this study.

### Indications

We diagnosed ALF based on the following criteria [7–9]: lab indication of jaundice progressing quickly with total bilirubin (TBil) ≥ 10 × upper limit of normal (ULN) or daily increasing data ≥ 17.1 μmol/L. Patients with ALF usually have hyperbilirubinemia and develop multiple organ dysfunction (MODS) after cardiac surgery. We divided the 56 patients into two groups: 14 patients who received BA treatment and 42 who received PEX treatment.

### Treatment approach

Vascular access was obtained via a double-lumen hemodialysis catheter introduced into the femoral, jugular or subclavian veins. Blood anticoagulation was controlled using unfractionated heparin (target clotting time of 140–200 s).

For the BA treatment device, PF 2000N (Gambro Dialysatoren GmbH) was used as the plasma filter, BS330 (Jafron Biomedical Co., Ltd, China) as the bilirubin absorption column (Jafron Biomedical Co., Ltd, China), HA330-II (Jafron, China) as the disposable hemoperfusion cartridge, and the Diapact CRRT system (Fresenius Medical Care, Germany) to perform the procedure; the following parameters were applied: blood flow rate 120–150 ml/min; plasma flow rate 30 ml/min; average duration of treatment 4 h; and average amount of plasma 6 L.

For the PEX treatment device, PF 2000N (Gambro Dialysatoren GmbH) was used as the plasma filter, and the Diapact CRRT system (Fresenius Medical Care, Germany) was used to perform the procedure; the following parameters were applied: blood flow rate 120–150 ml/min; plasma flow rate 25 ml/min; average duration of treatment 2 h; and average amount of plasma 3 L.

### Statistical analysis

Data analysis was performed using SPSS for Windows version 22 (IBM Corporation, Armonk, NY). Generally, continuous variables are presented as the mean ± standard deviation. Categorical variables are presented as absolute numbers and proportions (n, %). Differences in categorical variables were analyzed using the χ2 test. Differences in continuous variables were tested by t-test or the Mann–Whitney U-test. Some bias might exist in our study. Adjustment for indication bias was further assessed using a matched cohort. To overcome the bias in our study, we used a matched cohort of 28 patients (14 who received BA treatment and 14 who received PE treatment) with similar conditions. In the matched cohort, the PEX subjects were selected and matched for sex, age (± 2 years) and EuroSCORE II (± 1%). Differences were considered significant at p < 0.05.

## Results

Preoperative and intraoperative variables are listed in Table 1. The mean age of all the patients was 66.0 ± 10.6 years. Most of the patients were male (41/56, 73.2%) and had cardiac insufficiency (NYHA class III/IV, 55/56, 98.2%). The patients in the BA treatment group had higher risks of surgery mortality with higher EuroScore II scores than those in the PEX treatment group (p = 0.006). None of the patients had a history of chronic renal failure or liver disease. Baseline liver function and renal function were not different between the two groups. The type of cardiac operation showed no differences between the two groups. The mean cardiopulmonary bypass time and aortic cross-clamp were 225.9 ± 80.7 and 164.9 ± 60.7 min, respectively. The BA treatment group presented a longer bypass time and cross-clamp time, while there were no differences between the two groups.

**Table. 1:** Baseline and Characteristics

Variable		Bilirubin adsorption (n = 14)		Plasma exchange (n = 42)	P value
Age (year)		62.2 ± 14.9		67.2 ± 8.5	0.125
Gender (male, %)		10, 71.4%		31, 73.8%	0.862
Weight (kg)		64.4 ± 10.4		62.4 ± 8.6	0.477
NYHA class (n, %)					0.329
I		0		0	
II		0		1, 2.3%	
III		6, 42.8%		23, 54.8%	
IV		8, 57.2%		18, 42.9%	
EuroSCORE II		30.5 ± 17.5		20.9 ± 7.6	0.006
Previous Medical History (n,%)					
Acute Myocardial infarction		5, 35.7%		9, 21.4%	0.285
Diabetes Mellitus		7, 50.0%		11, 26.2%	0.099
Chronic Renal Failure		0		0	—
Hypertension		11, 78.6%		24, 26.2%	0.151
Liver Disease		0		0	—
Smoking		5, 35.7%		11, 26.2%	0.495
Type of cardiac operation (n,%)					0.890
CABG		1, 7.1%		4, 9.5%	
Valvular surgery		5, 35.7%		14, 33.3%	
CABG + Valvular surgery		4, 28.6%		13, 31.0%	
Aortic surgery		4, 28.6%		11, 26.2%	
CPB time		251.1 ± 79.7		218.1 ± 80.4	0.20
ACC time		187.8 ± 65.1		157.8 ± 58.2	0.12
Pre-operation liver function					
ALT		29.0 (13.4–58.7)		25.0.3 (17–51.3)	0.813
AST		34.1 (20.2–109.4)		30.5 (17.9–37.6)	0.348
GGT		102.8 ± 70.9		74.1 ± 38.2	0.225
TBil		23.3 ± 15.1		18.3 ± 9.9	0.157
DBil		10.3 ± 7.3		9.6 ± 6.4	0.715
LDH		230.9 ± 116.5		183.4 ± 83.4	0.176
Serum albumin		38.7 ± 4.8		38.8 ± 3.5	0.906
Pre-operation Serum Creatinine		115.9 ± 77.5		95.4 ± 53.8	0.276

Mean ± SD: Median (Interquartile range), CABG: Coronary artery bypass grafting, CPB: Cardiopulmonary bypass, CPB: Cardiopulmonary bypass, AST: Aspartate aminotransferase, CPB: Cardiopulmonary bypass, ALT: Alanine aminotransferase, GGT: Gamma-glutamyl transpeptidase, TBil: Total bilirubin, DBil: Direct bilirubin, LDH: Lactate dehydrogenase, NYHA: New York Heart Association

The baseline total bilirubin (TBil) level of all patients before treatment was 306.0 ± 100.7 μmol/l, and the direct bilirubin (DBil) level was 174.0 ± 68.3 μmol/l. Before adsorption or exchange, DBil (p = 0.040) levels were much higher in the BA treatment group than in the PEX treatment group. Compared to the plasma exchange treatment, the bilirubin adsorption treatment provided a statistically significant reduction in TBil (p = 0.016) and DBil (p = 0.036) levels. The details of the levels of TBil and DBil before and after BA/PE treatment are shown in Table 2.

**Table. 2:** Hepatorenal function after treatment

Variable		Bilirubin adsorption (n = 14)		Plasma exchange (n = 42)	P value
Before adsorption/exchange					
ALT		68.6 (40.3–139.5)		56.1 (40.3–82.0)	0.635
AST		83.6 (55.9–110.5)		91.3 (5.9–169.5)	0.669
GGT		103.8 ± 76.3		87.6 ± 60.7	0.422
TBil		329.8 ± 115.1		298.1 ± 95.6	0.311
DBil		206.2 ± 80.8		163.2 ± 60.9	0.040
LDH		555.5 (418.5–1142.2)		497.5 (164.7–636.2)	0.204
Serum Creatinine		123.7 ± 55.9		108.7 ± 38.4	0.265
BUN		15.8 ± 7.0		14.1 ± 5.7	0.367
C-creative protein		138.8 ± 52.1		142.6 ± 50.8	0.871
Child–Pugh Classification (n, %)					0.501
A		0		3, 7.1%	
B		7, 50.0%		21, 50.0%	
C		7, 50.0%		18, 42.9%	
After adsorption/exchange					
ALT		40.5 (31.3–66.8)		42.1 (32.9–86.8)	0.383
AST		67.0 (63.9–80.5)		76.3 (65.1–88.8)	0.372
GGT		81.7 ± 67.2		107.6 ± 90.2	0.331
TBil		187.8 ± 66.5		237.9 ± 196.6	0.016
DBil		106.1 ± 42.6		143.4 ± 71.5	0.036
LDH		418.6 ± 146.8		440.0 ± 170.0	0.675
Serum Creatinine		75.2 ± 15.3		90.8 ± 30.1	0.136
BUN		11.2 ± 2.9		12.4 ± 3.9	0.334
C-creative protein		106 (81.5–124.5)		112.0 (78.0–145.0)	0.393

BUN: Blood urea nitrogen, Mean ± SD: Median (Interquartile range), LDH: Lactate dehydrogenase, AST: Aspartate aminotransferase, ALT: Alanine aminotransferase, GGT: Gamma-glutamyl transpeptidase, TBil: Total bilirubin, DBil: Direct bilirubin

Serum aminotransferase levels reflecting hepatocyte cytolysis syndrome also significantly improved: aspartate aminotransferase (AST) and alanine aminotransferase (ALT) levels were both decreased after BA or PEX treatment (Table 2). Since the majority of the patients with AFL received renal replacement therapy before BA or PEX treatment for MODS, the baseline serum creatinine and blood urea nitrogen (BUN) levels were not very serious: the serum creatinine level was 112.3 ± 43.4 μmol/l, and the BUN level was 14.5 ± 6.0 mmol/l. After BA or PEX treatment, the serum creatinine level was 86.8 ± 27.8 μmol/l, and the BUN level was 12.1 ± 3.7 mmol/l (Table 2). The improvement in renal function indicated that both treatments could eliminate water-soluble toxic substances.

The in-hospital mortality was 85.7% (48/56) in the whole group, and the BA group had a trend toward a lower mortality than the PEX group (71.4% vs. 90.5%, P = 0.078). BA treatment showed better circulatory support, including lower risks of IABP (21.4% vs. 52.4%, p = 0.044), ECMO (21.4% vs. 50.0%, p = 0.061), reintubation (64.3% vs. 40.5%, p = 0.122) and ventricular arrhythmias (64.3% vs. 45.2%, p = 0.217). One of the main efficacy criteria of treatment therapy was its impact on maintaining homeostasis. BA treatment reduced the incidence of hepatic encephalopathy (35.7% vs. 71.4%, p = 0.017) and septic shock (35.7% vs. 52.4%, p = 0.280). The peak lac adsorption (p = 0.004) and vasoactive inotropic score (VIS) (p = 0.002) after treatment were both lower in the BA treatment group than in the PEX treatment group. Although the difference in the two groups was not statistically significant, there seems to be some practical difference in that the BA treatment group showed better clinical efficacy and outcomes. The detailed early outcomes are listed in Table 3.

**Table. 3:** Outcomes

Variable		Bilirubin adsorption (n = 14)	Plasma exchange (n = 42)	P value
Death (n, %)		10, 71.4%	38, 90.4%	0.078
CRRT (n, %)		12, 85.7%	30, 71.4%	0.285
IABP (n, %)		3, 21.4%	22, 52.4%	0.044
ECMO (n, %)		3, 21.4%	21, 50.0%	0.061
Re-intubation (n, %)		9, 64.2%	17, 40.5%	0.122
Septic shock (n, %)		5, 35.7%	22, 52.4%	0.280
Ventricular arrhythmias (n, %)		9, 64.2%	19, 45.2%	0.217
Hepatic encephalopathy (n, %)		5, 35.7%	30, 71.4%	0.017
Peak VIS after adsorption/exchange		51.5 ± 12.2	65.1 ± 15.6	0.002
Peak lac adsorption/exchange (mmol/L)		8.4 ± 2.1	10.6 ± 2.3	0.004

Lac: arterial lactate

After matching, a total of 28 patients were enrolled in the analysis (14 in the BA treatment group and 14 in the PEX treatment group). The in-hospital mortality was lower in the BA treatment group than in the PEX treatment group (71.4% vs. 100%, P = 0.049). The circulatory supports were similar between the two groups, including the usage of IABP, ECMO, reintubation and ventricular arrhythmias. The BA treatment group had advantages in maintaining homeostasis, reflecting a lower incidence of hepatic encephalopathy (35.7% vs. 92.9%, p = 0.002) and lower VIS score (P = 0.013) and lac levels (P = 0.045) after treatment. The BA treatment group also showed better efficacy in removing toxins and exhibited a statistically significant reduction in TBil, DBil, ALT, AST, serum creatinine, BUN and C-creative protein levels. The results of the matched cohort are listed in Table 4.

**Table. 4:** Outcomes and hepatorenal function in matched cohort

Variable		Bilirubin adsorption (n = 14)	Plasma exchange (n = 14)	P value
Death (n, %)		10, 71.4%	14, 100.0%	0.049
CRRT (n, %)		12, 85.7%	12, 85.7%	—
IABP (n, %)		3, 21.4%	8, 57.15%	0.060
ECMO (n, %)		3, 21.4%	3, 21.4%	—
Re-intubation (n, %)		9, 64.2%	10, 71.4%	0.500
Septic shock (n, %)		5, 35.7%	4, 28.6%	0.500
Ventricular arrhythmias (n, %)		9, 64.2%	6, 42.9%	0.225
Hepatic encephalopathy (n, %)		5, 35.7%	13, 92.9%	0.002
Peak VIS after adsorption/exchange		47.3 ± 5.9	62.9 ± 19.8	0.013
Peak lac adsorption/exchange (mmol/L)		8.4 ± 2.2	10.1 ± 2.1	0.045
After adsorption/exchange				
ALT		28.9 (19.4–64.0)	42.1 (32.9–94.0)	0.021
AST		58.0 (46.6–74.5)	76.3 (60.3–101.7)	0.012
GGT		69.0 (20.0–118.5)	79.0 (30.0–125.3)	0.667
TBil		162.9 ± 76.3	235.2 ± 82.9	0.024
DBil		90.4 ± 35.2	139.1 ± 53.5	0.009
LDH		394.5 ± 127.8	435.1 ± 161.7	0.467
Serum Creatinine		80.1 ± 11.9	107.8 ± 22.8	0.001
BUN		10.3 ± 2.8	14.5 ± 4.3	0.010
C-creative protein		83.5 ± 15.8	151.6 ± 49.2	0.018

## Discussion

The occurrence of hyperbilirubinemia after cardiac surgery has been observed for a long time, and despite demonstrable improvements in surgical techniques and perioperative care over the last decade, hepatic dysfunction remains a serious postoperative complication [10, 11]. Relevant factors for hyperbilirubinemia after cardiac surgery include [4, 12–14] the following: (1) hepatic ecchymosis due to high pressure of the right atrium; (2) nonpulsatile flow in CPB and its associated risk of regional malperfusion causing liver ischemic damage; (3) massive transfusion; (4) hemolysis caused by cardiotomy suction, membrane oxygenation and various other elements of CPB; and (5) postoperative infection. Different methods have been used to treat hyperbilirubinemia, such as the molecular adsorbent recirculating system (MARS), plasma exchange (PEX) and bilirubin adsorption (BA). It is still unclear which treatment strategy is more useful for patients with hyperbilirubinemia after cardiac surgery15. We presented a retrospective analysis of prospectively collected data that compared BA treatment with PEX treatment in hyperbilirubinemia after cardiac surgery. This study demonstrated the following: (I) BA treatment could be considered an effective strategy for the reduction of TBil and DBil levels in patients with postoperative hyperbilirubinemia. (II) Compared to PEX treatment, BA treatment could reduce in-hospital mortality and risks of poor outcomes.

Several studies have reported that the incidence of hyperbilirubinemia after cardiac surgery is between 10 and 40% [4, 16, 17], which has been consistent since the first report in 1967 [2]. In our center, the incidence of hyperbilirubinemia is 0.48%, which is similar to previous studies. Most patients suffered severe cardiac disease with NYHA class III/IV [18], and valvular surgery and CABG were the most common surgery types. These findings are similar to those of previous studies, and complicated valve surgery procedures caused more frequent postoperative hyperbilirubinemia [4, 12, 18–20]. Therefore, the severity and complexity of valve surgery might be important predictive factors for the incidence of postoperative hyperbilirubinemia.

When hyperbilirubinemia turned to acute liver failure, a large amount of endotoxin, cytokines and other pathogenic factors, especially those associated with albumin, accumulated in plasma. The combined toxin was difficult to pass through traditional blood purification treatments such as hemodialysis. These toxins play a key role in the development of liver failure and can cause hemodynamics and hepatorenal syndrome. It has been proven that short-term mortality depends on high levels of bilirubin [21], and low levels of bilirubin can facilitate hepatocyte regeneration. High levels of bile acids may induce apoptosis and cell necrosis of hepatocytes and retard hepatic regeneration [22]. In addition, bilirubin has neurotoxic and encephalopathic effects [23]. For these reasons, the removal of bilirubin seems to be an important therapeutic target. BA treatment and PEX treatment are both effective therapies for hyperbilirubinemia [24, 25]. Plasma exchange therapy can remove a variety of toxins, supply coagulation factors and regulate immune function. Bilirubin adsorption works through resin adsorbents, which have acceptable capacity for toxins such as bilirubin and cytokines. In our study, we found that BA treatment had a higher removal ability for TBil and DBil than PEX treatment, while the removal ability of ALT, AST and serum creatinine was similar between the two treatments. The following rate-limiting factors influenced the removal ability of albumin-binding toxins: [1] plasma ion strength and pH value [26]; [2] the possible loss of albumin due to its binding to the absorber columns [27]; and [3] the molar ratio of bilirubin to albumin [28]. The 20-fold higher molar ratio of serum bilirubin to albumin compared to the respective dialysate [26, 29] and the loss of albumin with time were due to its binding to the filter [27].

Our findings demonstrated that postoperative hyperbilirubinemia resulted in significantly increased in-hospital mortality, as the mortality was up to 85.7%, which was much higher than the reported early mortality between 19 and 25% [3, 18]. Patients in our study were all critical patients with severe congestive heart disease, and most of them were NYHA class III/IV. EuroSCORE II scores showed that these patients suffered huge risks of mortality and complications. Indeed, patients in our study had almost acute liver failure with MODS, and the mortality of MODS after cardiac surgery was reported to be up to 90% [5, 6]. Acute liver failure combined with MODS can cause disorder of the internal environment and hemodynamic instability. In this study, most patients suffered poor clinical outcomes, especially in the usage of IABP, ECMO, CRRT, etc. Almost all the patients died of MODS.

There have been limited studies regarding the optimal techniques for bilirubin removal, and no direct comparison exists between BA and PEX in patients after cardiac surgery. Recently, Chen X and his colleagues concluded that BA treatment was an effective and safe method for treating hyperbilirubinemia in patients after cardiac surgery [30]. Our study added evidence that BA treatment not only had a higher bilirubin removal ability but also could lower the mortality and risks of poor clinical outcomes in patients with hyperbilirubinemia after cardiac surgery. Moreover, PEX treatment requires a large amount of plasma or albumin, which could be associated with limitations regarding plasma and patients with rare blood types. BA treatment has an advantage in this aspect; it can adsorb bilirubin in a competitive binding manner compared to albumin. After the free bilirubin in plasma is adsorbed, the bilirubin bound to plasma albumin is partially dissociated and then adsorbed, and albumin and coagulation factors are protected in this way.

## Study limitations

This study was a retrospective study with limited patients in one center. Missing data for other possible factors, such as the amount of bleeding, postoperative central venous pressure and coagulation function, may limit our findings. We tried to compare two treatment therapies more clearly according to a matched cohort. However, factors that affect assignment to treatment and outcomes but that cannot be observed could not have been accounted for in the matched cohort. Moreover, after matching, the sample size was decreased, which may result in some statistical errors. Furthermore, the patients in the two groups were chosen from different periods, which may also cause some potential errors. These hidden biases might have remained in the matched cohort and led to statistical errors. In addition, the treatment timing for different therapies was not discussed in this study, and we thought a larger sample size and prospective studies would be needed to investigate this issue.

## Conclusion

Compared to PEX treatment, BA treatment had a higher bilirubin removal ability in patients with hyperbilirubinemia and could reduce the mortality and risks of poor clinical outcomes. BA treatment should be considered an effective treatment method for patients with higher TBil levels or DBil levels.

## Data Availability

The datasets used and/or analyzed during the current study are available from the corresponding author on reasonable request.

## Abbreviations

CABG: Coronary artery bypass grafting
CPB: Cardiopulmonary bypass
ACC: Aortic cross-clamp
AST: Aspartate aminotransferase
ALT: Alanine aminotransferase
GGT: Gamma-glutamyl transpeptidase
TBil: Total bilirubin
DBil: Direct bilirubin
LDH: Lactate dehydrogenase
NYHA: New York Heart Association
BUN: Blood urea nitrogen
Lac: Arterial lactate
CRRT: Continuous renal replacement therapy
IABP: Intra-aortic balloon pump
ECMO: Extracorporeal membrane oxygenation
